# The Effect of Low-Carbohydrate Diet on Glycemic Control in Patients with Type 2 Diabetes Mellitus

**DOI:** 10.3390/nu10060661

**Published:** 2018-05-23

**Authors:** Li-Li Wang, Qi Wang, Yong Hong, Omorogieva Ojo, Qing Jiang, Yun-Ying Hou, Yu-Hua Huang, Xiao-Hua Wang

**Affiliations:** 1 School of Nursing, Medical College, Soochow University, Suzhou 215006, China; wanglili83476@suda.edu.cn (L.-L.W.); xuweipan@whu.edu.cn (Q.W.); 20175231003@stu.suda.edu.cn (Y.H.); jiangqing2015@suda.edu.cn (Q.J.); houyunying@suda.edu.cn (Y.-Y.H.); 2 Department of Adult Nursing and Paramedic Science, University of Greenwich, London SE9 2UG, UK; o.ojo@greenwich.ac.uk; 3 Medical College, Soochow University, Suzhou 215006, China; Huangyuhua@suda.edu.cn

**Keywords:** diabetes mellitus, diet, carbohydrate, blood glucose, HbA1c, fasting blood glucose, postprandial blood glucose

## Abstract

Objective: In China, a low-fat diet (LFD) is mainly recommended to help improve blood glucose levels in patients with type 2 diabetes mellitus (T2DM). However, a low-carbohydrate diet (LCD) has been shown to be effective in improving blood glucose levels in America and England. A few studies, primarily randomized controlled trials, have been reported in China as well. Method: Firstly, we designed two ‘six-point formula’ methods, which met the requirements of LCD and LFD, respectively. Fifty-six T2DM patients were recruited and randomly allocated to the LCD group (*n* = 28) and the LFD group (*n* = 28). The LCD group received education about LCD’s six-point formula, while the LFD group received education about LFD’s six-point formula. The follow-up time was three months. The indicators for glycemic control and other metabolic parameters were collected and compared between the two groups. Results: Forty-nine patients completed the study. The proportions of calories from three macronutrients the patients consumed met the requirements of LCD and LFD. Compared to the LFD group, there was a greater decrease in HbA1c level in the LCD group (−0.63% vs. −0.31%, *p* < 0.05). The dosages of insulin and fasting blood glucoses (FBG) in the third month were lower than those at baseline in both groups. Compared with baseline values, body mass index (BMI) and total cholesterol (TC) in the LCD group were significantly reduced in the third month (*p* < 0.05); however, there were no statistically significant differences in the LFD group. Conclusions: LCD can improve blood glucose more than LFD in Chinese patients with T2DM. It can also regulate blood lipid, reduce BMI, and decrease insulin dose in patients with T2DM. In addition, the six-point formula is feasible, easily operable, and a practical educational diet for Chinese patients with T2DM.

## 1. Introduction

Dietary intervention is a strategy to manage diabetes mellitus (DM) [1], as it can reduce the burden on islet cells and thus improve blood glucose levels, lipid profiles, and cognitive status [2,3,4]. However, good adherence to diabetic diets is the premise of diet therapy. In China, a low-fat diet (LFD) is mainly recommended to help improve blood glucose levels in patients with type 2 diabetes mellitus (T2DM) [5]. Studies have shown that LFD could reduce glycated hemoglobin (HbA1c) by as much as 0.8–2.8% [6,7,8].

On the other hand, a low-carbohydrate diet (LCD) is a dietary strategy that refers to carbohydrate intake of between 30–200 g/day or calories from carbohydrates/total calories of <45%, supplementing instead with fat or protein [9]. This has been found to be effective in the treatment of obesity, and apart from significantly reducing weight, it can also effectively improve blood lipid and insulin resistance [10]. In recent years, the American Diabetes Association and Diabetes UK have both confirmed the effectiveness of LCD in reducing weight, improving blood glucose, and regulating blood lipid in patients with DM [11,12]. In Japan, Yamada [13] reported that HbA1c and triglyceride (TG) levels in patients with T2DM decreased significantly in the LCD group without calorie-restriction, compared to the LFD group with calorie-restriction. This indicates that LCD made patients with DM have less desire to eat due to a feeling of satiety. However, only limited studies relating to the use of LCD in patients with DM, especially randomized controlled trials, have been reported in China.

Based on research evidence, only 29.8% of Chinese patients with T2DM comply with a diabetic diet advised by their doctors and dietitians [14]. In addition, we found that certain types of foods were strictly limited and patients with DM were finding it hard to understand the caloric values of foods consumed, thus making it difficult to adhere to the diet. Thus, it is necessary to develop an easy and more effective method to support these patients. Firstly, we designed the ‘six-point formula’ to help patients master LCD and LFD. We then let them record details of their diets and hand over to us the task of calculating the caloric values of foods. Based on this, we explored the effect of two DM diets (LCD and LFD) on hyperglycemia.

## 2. Materials and Methods

### 2.1. Subjects

Participants with T2DM were recruited from the community and the First Affiliated Hospital of Soochow University. The inclusion criteria were the following: Patients older than 18 years, had been diagnosed with T2DM, had no change in oral antidiabetic drugs or insulin in half a month before the intervention, were able to communicate, had volunteered to participate in this study, and are able to provide informed consent. Those excluded were patients who ate nuts regularly (≥4 day/week ) [15]; were allergic to food, especially nuts; had difficulty in chewing nuts (such as those with few teeth); received other dietary interventions or had severe conditions including indigestion, heart failure, renal failure, malignant tumours, severe cerebrovascular disease, ketosis, digestive dysfunction, liver dysfunction or severe gallbladder and pancreatic diseases; and those whose fasting blood glucose (FBG) were more than 16.7 mmol/L [16] during the interventions.

### 2.2. Study Design

This study is a prospective, single-blind randomized controlled trial (RCT) performed between December 2015 to December 2016. The recruited patients were randomly allocated to receive either LCD or LFD using a table of random numbers. Before the intervention, all subjects underwent a one-week [17] washout period to diminish the effect of background diets on the study. The patients were blinded when assigned to groups. This study followed the Declaration of Helsinki and the Guidelines for Good Clinical Practice and was approved by the ethics committee of the First Affiliated Hospital of Soochow University (No. 2015106). All enrolled patients signed a consent form.

### 2.3. Sample Size Calculation

Evidence from the literature showed that changes in the HbA1c level for six months were 0.6 ± 0.5% in the LCD group and 0.2 ± 0.5% in the calorie-restricted group [13]. Therefore, we calculated 25 patients for each group, with α = 0.05 and power = 0.80. In view of the sample loss of 10%, the number for each group was 28. Finally, we recruited 28 patients for each group in the study.

### 2.4. Biochemical Parameters and Analyses

Glycated hemoglobin provides an estimate of glycemic control for the past three months and is predictive of clinical outcomes [18]. HbA1c was measured at baseline and at the end of the third month. Blood samples were obtained to measure HbA1c at the nursing School of Soochow University and measured by high-performance liquid chromatography using Afinion AS100 Analyzer (Alere, Inc., Shanghai, China) in the molecular laboratory of the nursing school of Soochow University. Fasting blood glucose (FBG) and postprandial 2-h blood glucose levels were measured by collecting the peripheral blood from fingers using rapid glycaemic apparatus by patients once a week at home.

Fasting blood samples were also collected for various biochemical assays, including total cholesterol (TC), performed as per the experimental protocol in hospitals.

Hypoglycemic episodes in this study were determined by the self-reported hypoglycemic symptoms of patients with or without a measured plasma glucose concentration <70 mg/dL (3.9 mmol/L) or only a measured plasma glucose concentration <70 mg/dL (3.9 mmol/L). Therefore, all episodes of abnormal low plasma glucose concentration that exposed the individual to potential harm and other clinical incidents, including severe hypoglycemia, documented symptomatic hypoglycemia, asymptomatic hypoglycemia, probable symptomatic hypoglycemia and relative hypoglycemia referred to the self-reported hypoglycemic symptoms of patients without a measured plasma glucose concentration <70 mg/dL (3.9 mmol/L), were considered [19]. In this study, the modification of hypoglycemic agents referred to change in the quantities of insulin dosages the participants used at baseline and in the third month. Researchers collected data of modification of hypoglycemic agents at every follow up.

### 2.5. Anthropometric Measurements

Body mass index (BMI) was calculated as weight (in kilograms) divided by height (in meters squared). At baseline and in the third month, the weight and height of patients were measured by a unified measuring device at the nursing school of Soochow University.

### 2.6. Diet Record

Patients maintained a diet record, including a detailed diet of any day over the weekend and two working days. The composition of the diets was calculated using the Chinese CDC nutrition calculator V2.63 software (Development team of Fei Hua nutrition software, Beijing, China) and the quantities and distributions of energy from three macronutrients intake was determined. This also enabled an understanding of the patients’ dietary adherence.

### 2.7. Intervention

**Figure 1:**
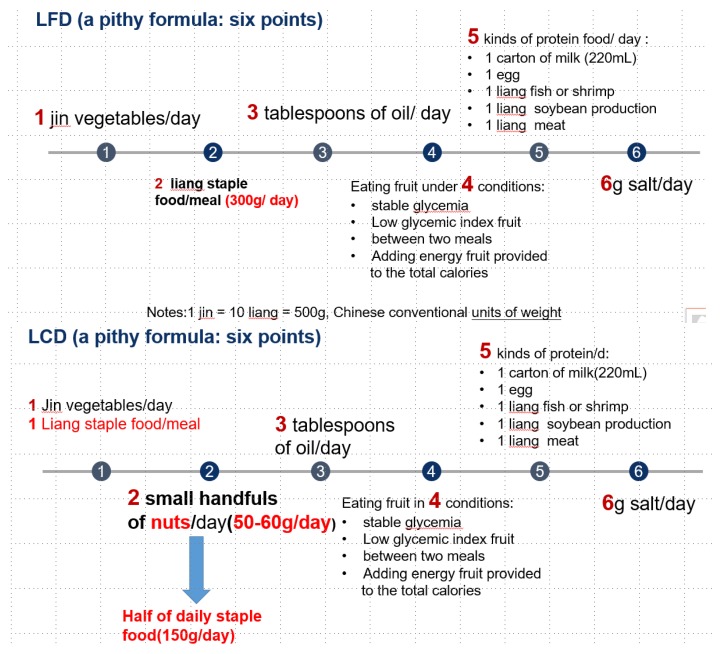
The detailed contents of the six-point formula of two groups. Notes: 1 jin = 10 liang = 500 g, Chinese conventional units of weight. Staple food/meal refers to foods rich in carbohydrates, mainly three kinds of steamed bread, noodles and rice in China. LFD: Low-fat diet; LCD: Low-carbohydrate diet.

Firstly, our team developed a preliminary dietary education handbook for patients with T2DM based on evidence from literature and guidelines regarding T2DM dietary management [5,20]. Secondly, two endocrinologists, four diabetic nurse specialists, and one dietician reviewed and modified the handbook. Finally, five T2DM inpatients of different ages and educational levels reviewed the handbook to ensure that patients with T2DM understood it and that it could help improve their dietary adherence. The major content of the handbook was a concise formula that included six points. Detailed contents of the six-point formula are shown in Figure 1. Other educational contents about foods included how to distinguish vegetables and staple food (such as potato and broad bean); ways to cook food; and symptoms, prevention and treatment of hypoglycemia.

In the one-on-one education session, the researcher and the patients reviewed the handbook. Using the LCD handbook, the researcher focused on instructing patients to restrict intake of staple food/meal (1 Liang) per day in the LCD group. The reduced staple food/meal was replaced by consuming 60 g/day nuts for males and 50 g/day for females, respectively. Nuts were uniformly purchased, weighed, vacuum-packed, and distributed every two weeks.

For patients in the LFD group, we provided participants with a handbook about LFD, and instructed them on a pithy formula of six points.

Follow up was conducted once a week in the first month of the intervention and once every two weeks in the second and third months. The duration of follow up was about 10 min. The main focus of the follow-up was to review the patients’ compliance to the diet program and to support them to adhere to it (in patients with poor compliance). It also involved collecting data of the modification of hypoglycemic agents and the occurrence of hypoglycemia. If a patient’s diet did not meet the requirements of the dietary program in the intervention period, they were excluded from the study.

### 2.8. Statistical Analysis

Statistical analyses were performed using SPSS 18.0 software (SPSS, Inc., Chicago, IL, USA). For continuous variables, the results were described as the mean ± standard deviation (SD) and comparisons were performed using Independent Samples *t*-test, paired samples *t*-test or the Wilcoxon rank-sum test. For categorical variables, the results were presented as frequency (percentages); comparisons between groups were made using the Chi-squared test or Fisher’s exact test. The trends in the FBG and postprandial 2 h blood glucose in two groups during the intervention were described by the fold line diagram. Intention-To-Treat (ITT) of HbA1c was performed to ensure the reliability of research results. A *p* value of < 0.05 was considered statistically significant.

## 3. Results

### 3.1. Study Participants

**Figure 2:**
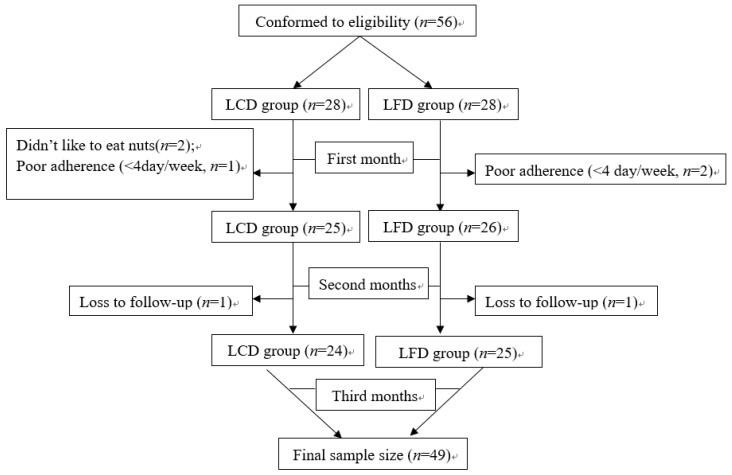
Flow diagram of the patients.

On the basis of inclusion and exclusion criteria, 56 T2DM participants were recruited and randomly allocated to the LCD group (*n* = 28) and the LFD group (*n* = 28). Four participants in the LCD group and three participants in the LFD group withdrew from the study. In the LCD group, two participants didn’t like nuts, one showed poor adherence (<4 day/week, and one was lost during follow-up. In the LFD group, two showed poor adherence to the diet program (<4 day/week) and one was lost during follow-up. Finally, the data of 24 in the LCD group and 25 in the LFD group were analyzed (Figure 2). The mean age of patients were (63.94 ± 10.79) years and 26 (53.1%) were men. The general characteristics of the enrolled participants in each group are shown in Table 1 There were no statistically significant differences in any of the parameters between the two groups (*p* > 0.05).

### 3.2. Dietary Adherence

#### 3.2.1. Comparison of Dietary Adherence

Dietary adherence was assessed mainly from two aspects: the days of adherence to the dietary program per week and macro-nutrient allocation and their quantities. The Wilcoxon rank-sum test was performed to compare dietary compliance in the two groups (LCD versus LFD). The result showed that there was no difference in self-reported dietary compliance per week (*p* > 0.05, Table 2).

#### 3.2.2. Proportions of Calories from Three Macronutrients the Patients Consumed

**Figure 3:**
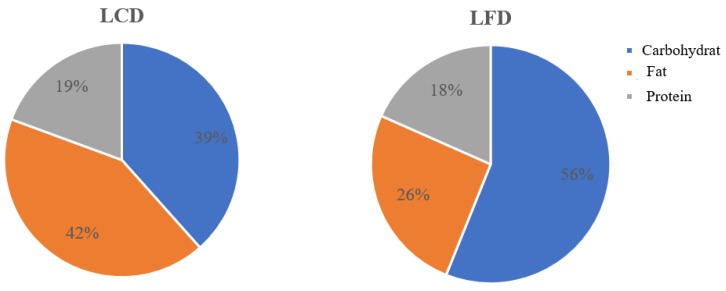
The percentage of the calories from carbohydrates (39%) met the standard of LCD (<45%) in the LCD group, while the 26% calories from fat met the standard of LFD. LCD: Low-carbohydrate diet; LFD: Low-fat diet

Prior to the intervention, the total energy and the proportions of calories from the three major nutrients were not significantly different between the two groups (LCD versus LFD). After the intervention, compared to the LFD group, the calories from carbohydrates decreased, while those from fat significantly increased in the LCD group (*p* < 0.05). In addition, the percentage of calories from carbohydrates (39%) met the standard of LCD (<45%). The 26% of calories from fat met the standard of LFD, while the calories from protein were almost similar in the two groups (*p* > 0.05, Table 3) (Figure 3).

### 3.3. Effect of LCD on Glycemic Control

#### Glycated Hemoglobin

Compared to the baseline, HbA1c levels in both the LCD group and LFD group decreased significantly (0.63 ± 1.18% and 0.31 ± 0.70%), respectively. At the baseline, HbA1c levels were not significantly different between the two groups. However, after the intervention, HbA1c levels in the LCD group decreased significantly (*p* < 0.05, Table 4), when compared to the LFD group. The Intention-To-Treat (ITT) in relation to HbA1c levels was performed to ensure the stability of the above results. The ITT results were found to be in agreement with the earlier findings (Table 5).

### 3.4. Fasting Blood Glucose

#### 3.4.1. Changing Trends of Fasting Blood Glucose

**Figure 4:**
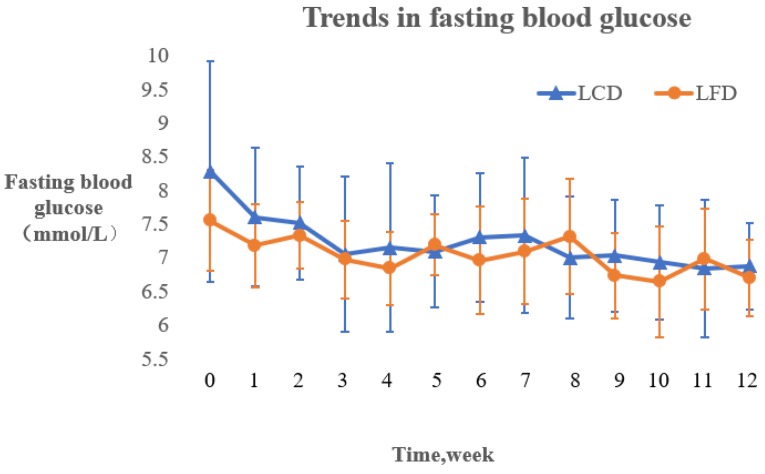
The changing trends of the FBG in the LCD and LFD Groups. FBG: fasting blood glucoses

The changing trends of the FBG in the two groups during the intervention are described by the fold line diagram (Figure 4). The results showed that the change of FBG in the LCD group decreased significantly for the first four weeks and then decreased steadily after the fourth week. In contrast, the FBG in the LFD group demonstrated dynamic fluctuation, although it was lower than the baseline value.

#### 3.4.2. Comparison of Fasting Blood Glucose levels

Compared to the baseline, FBG levels of the two groups significantly improved (*p* < 0.01). But the differences between the two groups with respect to FBG was not statistically significant (*p* > 0.05) (Table 6).

### 3.5. Postprandial Two-Hour Blood Glucose

#### 3.5.1. Trends in Postprandial Two-Hour Blood Glucose

**Figure 5:**
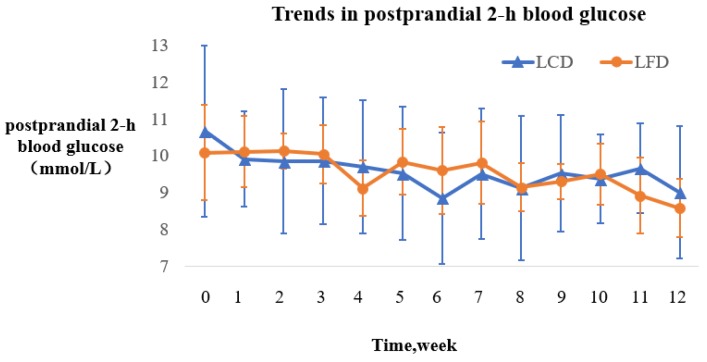
The changing trends of the postprandial 2-h blood glucose in the LCD and LFD Groups.

The changing trends of the postprandial 2-h blood glucose of the two groups during the intervention are described by the fold line diagram (Figure 5.). Both groups showed fluctuation in this indicator.

#### 3.5.2. Comparison of Postprandial Two-Hour Blood Glucose

Compared to the baseline, the postprandial 2-h blood glucose in the two groups improved significantly (*p* < 0.01). However, there was no significant difference between the two groups (*p* > 0.05) (Table 7).

### 3.6. Effect of LCD on Other Metabolic and Anthropometric Indicators

Compared to the baseline, body mass index (BMI) and total cholesterol (TC) in the LCD group improved significantly in the third month (*p* < 0.05). However, there were no similar results in the LFD group. After the intervention, the metabolic indicators were not significantly different between the two groups (Table 8).

### 3.7. Hypoglycemia and Medication Changes

#### 3.7.1. Frequency of Hypoglycemia

The frequencies of hypoglycemia during the three-month period in the two groups showed no significant differences (*p* > 0.05), before and after the intervention. In addition, there were no significant differences (*p* > 0.05) between the two groups before and after the interventions (Table 9)

#### 3.7.2. The Dosages of Insulin Used

When compared to the baseline, the dosage of insulin used in the two groups decreased significantly after the intervention (*p* < 0.05, Table 10), although there was no significant difference between the two groups (*p* > 0.05).

#### 3.7.3. The Changes of Other Antidiabetic Drugs

There was no significant difference between the two groups in the third month (*p* > 0.05, Table 11).

## 4. Discussion

The use of LCD in human nutrition and health is a dietary strategy that ensures that carbohydrate intake is restricted. However, in a Chinese dietary plan, most staple foods have high glycemic index [20,21]. Therefore, it would seem that LCD may not be accepted easily among Chinese patients with diabetes mellitus (DM). In consideration, we initially designed the ‘six-point formula’ to help patients improve dietary adherence. We found that the participants showed good adherence to the intervention, and no significant difference with respect to dietary adherence between two groups (LCD versus LFD) was observed. The proportions of energy provided by the three macronutrients met the requirements of LCD and LFD. It was indicated that the ‘six-point formula’ of the DM diet was feasible for Chinese T2DM patients.

### 4.1. Effect of LCD on Glycemic Control

High levels of HbA1c, FBG, and postprandial 2h blood glucose levels are some of the most difficult challenges faced by patients with T2DM and these parameters could be used as the main indicators to establish glycemic control [5].

HbA1c levels can reflect blood glucose levels in 2~3 months before blood extraction and long-term glycemic control of patients [5]. The result of this study showed that HbA1c levels in LCD (8.5%) decreased significantly (*p* < 0.05) compared to that in LFD (4%). The reason might be due to the decreased level of high glycemic index foods, the total amount of foods rich in carbohydrates, and the increased intake of nuts, which could help improve hyperglycemia and insulin sensitivity [22,23,24]. Yamada et al. [13] showed that HbA1c levels were significantly decreased by as much as 7.9% in the LCD group and by only 2.6% in the calorie-restricted group. Mayer et al. [25] also found LCD led to a relative improvement in HbA1c than LFD. However, some studies have shown that LFD could decrease HbA1c by 0.8–2.08% [7,8]. These values were less than the result of our study, which might be due to the effect of the ‘six-point formula’ that was simple and easy to remember, helped patients master the methods of the DM diet better, and improved dietary compliance and hyperglycemia. 

Fasting blood glucose and postprandial 2-h blood glucose are important indicators for the diagnosis and monitoring of DM [5]. The fold line diagram in this study showed that FBG significantly decreased during the first four weeks in the two groups. While FBG steadily decreased in the LCD group, there was dynamic fluctuation after the initial first month in the LFD group. A reason for the same might be that the patients in the two groups showed keen interest in the ‘six-point formula’ at the beginning of the intervention, which helped improve their dietary adherence and promote FBG control. In addition, nuts could stabilize blood glucose levels [23,26,27], which may have contributed to the steady decrease of FBG in the LCD group. Postprandial 2h blood glucose obviously decreased in the LCD group, which might have resulted from its relationship to limited carbohydrates [20,22].

### 4.2. Other Metabolic Indicators

Nuts are high-fat diets with high-energy levels, but they do not increase the weight of patients [27] because they increase a feeling of satiety and lead to a strong dietary compensation effect [28]. In addition, energy absorption efficiency of the nuts is low and the total energy does not increase [28]. This study further confirmed that BMI in the LCD group decreased. The result is in agreement with the results of Li et al. [23] and Barbour et al. [29].

Diabetes is significantly related to dyslipidemia [5]. While we pay attention to blood glucose levels, it is also necessary to regulate blood lipids. Lovejoy et al. [30] found that TC level in diets enriched in almonds was lower by 21%. Our study found that the TC level decreased significant by 7.4% in the LCD group, which might be related to the effect of some ingredients of the nuts consumed [27].

### 4.3. Hypoglycemia and Medication Changes

We found that the insulin dose used by patients in the LCD group during the intervention period decreased, consistent with a study by Westman et al. [31], which found that patients could reduce or terminate the use of hypoglycemic agents by controlling the intake of carbohydrates. But there were no significant differences between group comparisons.

In this study, hypoglycemia is used as a safety indicator. Although there was no statistical change in the frequency of hypoglycemia in within-group comparison and no difference in between-group comparison, the frequencies of hypoglycemia were reduced in the two groups.

## 5. Limitation

There are some limitations to the study. Firstly, the method used to evaluate the energy intake of food may not have been robust enough. At the baseline, we obtained data of caloric intake from patients’ memories, which meant that it was probably underestimated. Secondly, measurement differences might exist in FBG and postprandial 2-h blood glucose levels, which were measured at home by the patients themselves using different blood glucose meters. Thirdly, the prolonged effect of LCD on the prognosis of DM was not observed due to short follow-up time. Finally, a control group without a treatment was not considered in the study design.

## 6. Conclusions

LCD can improve blood glucose more than LFD in Chinese patients with T2DM. It can also regulate blood lipids, reduce BMI, and decrease insulin doses in patients with T2DM. In addition, the six-point formula is feasible, easily operable, and is a practical educational diet for Chinese patients with T2DM.

## Figures and Tables

**Table 1:** Baseline characteristics.

Variables		LCD (*n* = 24)	LFD (*n* = 25)	*t*/χ^2^	*p*
		$\bar{x}$ ± SD/*n* (%)	$\bar{x}$ ± SD/*n* (%)		
**Demographic data**					
Age, years		66.79 ± 9.12	61.20 ± 11.71	1.860 ^a^	NS
Gender, Male		13 (54.2)	13 (52.0)	0.023 ^b^	NS
Marital Status	Married	23 (95.8)	22 (88)	1.728 ^c^	NS
	Unmarried	0 (0)	1 (4.0)		
	Widowhood	1 (4.2)	2 (8.0)		
Education level, years		9.63 ± 4.10	8.36 ± 3.12	1.219 ^a^	NS
Occupation status	On the job	4 (16.7)	10 (40.0)	3.267 ^b^	NS
	Retirement	20 (83.3)	15 (60.0)		
Residential status	Living by oneself	1 (4.2)	4 (16.0)	3.733 ^c^	NS
	Living with spouse	21 (87.4)	19 (76.0)		
	Living with children	1 (4.2)	2 (8.0)		
	Living with mother	1 (4.2)	0 (0)		
Medical insurance, No		1 (4.2)	1 (4.0)	0.001 ^b^	NS
Family Support	Value	19 (79.2)	14 (56.0)	2.988 ^b^	NS
	Ordinary	5 (20.8)	11 (44.0)		
Exercise	Never exercise	1 (4.2)	2 (8.0)	0.400 ^c^	NS
	Never regular exercise	11 (45.8)	12 (48.0)		
	Regular exercise	12 (50.0)	11 (44.0)		
**Clinical data**					
Smoking, yes		2 (8.3)	5 (20.0)	1.361 ^d^	NS
SBP, mmHg		131.42 ± 10.89	130.84 ± 14.83	0.155 ^a^	NS
DBP, mmHg		77.54 ± 10.48	76.40 ± 10.43	0.382 ^a^	NS
Family history of diabetes, yes		12 (50.0)	9 (36.0)	0.980 ^b^	NS
Diabetes duration, years		12.79 ± 6.49	9.10 ± 6.52	1.985 ^a^	NS
Oral antilipemic agents, yes		8 (33.3)	11 (44.0)	0.587 ^a^	NS
Oral antidiabetic drugs or/and insulin		22 (91.7)	22 (88.0)	- ^d^	NS
Complications, yes		9 (37.5)	5 (20.0)	1.838 ^b^	NS
Accompanying diseases, yes		17 (70.8)	19 (76.0)	0.168 ^b^	NS

*p* value for comparison between treatments diets by Independent Samples *t*-test or Chi-square test. ^a^ *t*-test; ^b^ Chi-square test; ^c^ Likelihood Ratio; ^d^ Fisher’s Exact Test. NS: Differences are not significant; SBP: Systolic blood pressure; DBP: Diastolic blood pressure.

**Table 2:** Comparison of dietary adherence between the two groups.

	LCD (*n* = 24)	LFD (*n* = 25)	Z	*p*
4 d/W	3 (12.5)	7 (28.0)	4.449	NS
5~6 d/W	7 (29.2)	10 (40.0)		
7 d/W	14 (58.3)	8 (32.0)		

*p* value for comparison by Wilcoxon rank-sum test. *Z:* Wilcoxon rank-sum test; NS: Differences are not significant.

**Table 3:** Comparison of the calories from three macronutrients consumed by the patients.

	Variables	LCD (*n* = 24)	LFD (*n* = 25)	*T*	*p*
Baseline	Total calorie intake/day	1796.0 ± 186.6	1768.8 ± 138.7	0.421	NS
	Carbohydrate-calorie (Kcal)	948.8 ± 130.9	922.5 ± 145.1	0.485	NS
	Fat-calorie (Kcal)	538.9 ± 92.4	542.0 ± 94.8	−0.084	NS
	Protein-calorie (Kcal)	306.6 ± 56.7	303.3 ± 41.8	0.166	NS
3rd month	Total calorie intake/day	1808.0 ± 190.7	1731.5 ± 109.6	1.257	NS
	Carbohydrate-calorie (Kcal)	695.2 ± 106.6	970.2 ± 101.1	−6.747	<0.001 **
	Fat-calorie (Kcal)	763.1 ± 99.1	442.8 ± 52.0	10.320	<0.001 **
	Protein-calorie (Kcal)	350.3 ± 64.4	317.4 ± 52.0	1.433	NS

*p* value for comparison by Independent Samples *t*-test. ** *p* < 0.01 NS: Differences are not significant.

**Table 4:** Comparison of glycated hemoglobin (%) between the two groups.

Study Period	LCD (*n* = 24)	LFD (*n* = 25)	*t*	*p*
Baseline	7.43 ± 1.39	7.79 ± 1.20	−0.971	NS
3rd month	6.80 ± 0.83	7.48 ± 1.15	−2.350	0.023 *
MD	0.63 ± 1.18	0.31 ± 0.70	-	-
*t*	2.601	2.213	-	-
*p*	0.016 *	0.037 *	-	-

*p* value for comparison by Independent Samples *t*-test or paired samples *t*-test. * *p* < 0.05. NS: Differences are not significant.

**Table 5:** Comparison of glycated hemoglobin (%) between the two groups in ITT.

Study Period	LCD (*n* = 28)	LFD (*n* = 28)	*T*	*p*
Baseline	7.39 ± 1.29	8.16 ± 1.59	−1.994	NS
3rd month	6.85 ± 0.79	7.89 ± 1.63	−3.017	0.004 **
MD	0.54 ± 1.12	0.28 ± 0.67	-	-
*t*	2.556	2.194	-	-
*p*	0.017 *	0.037 *	-	-

*p* value for comparison by Independent Samples *t*-test or paired samples *t*-test. * *p* < 0.05; ** *p* < 0.01; ITT: Intention-To-Treat; NS: Differences are not significant.

**Table 6:** Comparison of fasting blood glucose (mmol/L) between the two groups.

Study Period	LCD (*n* = 24)	LFD (*n* = 25)	*t*	*p*
Baseline	8.28 ± 1.64	7.55 ± 0.75	1.469	NS
3rd month	6.87 ± 0.65	6.70 ± 0.57	0.793	NS
*t*	4.873	3.889	-	-
*p*	<0.001 **	0.003 **	-	-

*p* value for comparison by Independent Samples *t*-test or paired samples *t*-test. ** *p* < 0.01. NS: Differences are not significant.

**Table 7:** Comparison of postprandial 2-h blood glucose (mmol/L) in the groups.

Study Period	LCD (*n* = 24)	LFD (*n* = 25)	*t*	*p*
Baseline	10.67 ± 2.33	10.08 ± 1.29	0.818	NS
3rd month	9.00 ± 1.80	8.58 ± 0.80	0.761	NS
*t*	4.690	3.786	-	-
*p*	<0.001 **	0.003 **	-	

*p* value for comparison between treatments diets by Independent Samples *t*-test or paired samples *t*-test. ** *p* < 0.01. NS: Differences are not significant.

**Table 8:** Comparison of other metabolic indicators between the two groups.

Variables	Study Period	LCD (*n* = 24)	LFD (*n* = 25)	*t*	*p*
BMI (Kg/m^2^)	Baseline	24.29 ± 3.36	24.62 ± 5.17	−0.261	NS
	3rd month	23.52 ± 2.70	23.47 ± 3.11	0.060	NS
	*t*	2.756	1.235	-	-
	*p*	0.011 *	NS	-	-
TC (mmol/L)	Baseline	4.85 ± 0.87	4.55 ± 1.04	1.101	NS
	3rd month	4.49 ± 0.86	4.63 ± 0.99	−0.521	NS
	*t*	2.540	−0.363	-	-
	*p*	0.018 *	NS	-	-

*p* value for comparison by Independent Samples *t*-test or paired samples *t*-test. * *p* < 0.05; BMI: Body mass index; TC: total cholesterol. NS: Differences are not significant.

**Table 9:** Comparison of the frequencies of hypoglycemia between the two groups.

Time	LCD (*n* = 24)	LFD (*n* = 25)	*t*	*P*
Baseline	0.21 ± 0.59	0.52 ± 0.77	−1.596	NS
3rd month	0.04 ± 0.20	0.36 ± 0.86	−1.798	NS
*t*	1.282	0.778	**-**	**-**
*p*	NS	NS	**-**	**-**

*p* value for comparison by Independent Samples *t*-test or paired samples *t*-test. NS: Differences are not significant.

**Table 10:** Comparison of insulin dose (insulin unit, IU) between the two groups.

Times	LCD (*n* = 7)	LFD (*n* = 13)	*t*	*P*
Baseline	31.14 ± 16.38	29.00 ± 12.27	0.332	NS
3rd month	28.29 ± 13.74	26.62 ± 11.20	0.294	NS
*t*	2.765	3.023	-	-
*p*	0.033 *	0.011 *	-	-

*p* value for comparison by paired samples *t*-test. * *p* < 0.05. NS: Differences are not significant.

**Table 11:** Comparison of other antidiabetic drugs between the two groups.

	LCD (*n* = 23)	LFD (*n* = 11)	χ^2^	*p*
No change	20 (87.0%)	11 (100%)	2.482	NS
Reduction	2 (8.7%)	0 (0)		
Addition	1 (4.3%)	0 (0)		

*p* value for comparison between treatments diets by Chi-square test. NS: Differences are not significant.
